# Changes in fetal mannose and other carbohydrates induced by a maternal insulin infusion in pregnant sheep

**DOI:** 10.1186/2049-1891-5-28

**Published:** 2014-05-22

**Authors:** Laura D Brown, Stephanie R Thorn, Alex Cheung, Jinny R Lavezzi, Frederick C Battaglia, Paul J Rozance

**Affiliations:** 1 Perinatal Research Center, Division of Neonatology, Department of Pediatrics, University of Colorado Denver School of Medicine, Aurora, CO, USA; 2 Center for Women’s Health Research, University of Colorado Denver School of Medicine, Aurora, CO, USA

**Keywords:** Fructose, Glucose, Inositol, Insulin, Mannose, Pregnancy

## Abstract

**Background:**

The importance of non-glucose carbohydrates, especially mannose and inositol, for normal development is increasingly recognized. Whether pregnancies complicated by abnormal glucose transfer to the fetus also affect the regulation of non-glucose carbohydrates is unknown. In pregnant sheep, maternal insulin infusions were used to reduce glucose supply to the fetus for both short (2-wk) and long (8-wk) durations to test the hypothesis that a maternal insulin infusion would suppress fetal mannose and inositol concentrations. We also used direct fetal insulin infusions (1-wk hyperinsulinemic-isoglycemic clamp) to determine the relative importance of fetal glucose and insulin for regulating non-glucose carbohydrates.

**Results:**

A maternal insulin infusion resulted in lower maternal (50%, *P* < 0.01) and fetal (35-45%, *P* < 0.01) mannose concentrations, which were highly correlated (r^2^ = 0.69, *P* < 0.01). A fetal insulin infusion resulted in a 50% reduction of fetal mannose (*P* < 0.05). Neither maternal nor fetal plasma inositol changed with exogenous insulin infusions. Additionally, maternal insulin infusion resulted in lower fetal sorbitol and fructose (*P* < 0.01).

**Conclusions:**

Chronically decreased glucose supply to the fetus as well as fetal hyperinsulinemia both reduce fetal non-glucose carbohydrates. Given the role of these carbohydrates in protein glycosylation and lipid production, more research on their metabolism in pregnancies complicated by abnormal glucose metabolism is clearly warranted.

## Background

The importance of non glucose carbohydrates for normal fetal and neonatal development is becoming increasingly recognized, especially for mannose and inositol [1-4]. Carbohydrates not only serve as substrates for the glycolytic pathway, but also are critical for glycoprotein formation [5], phospholipid and glycerol production [2,6], and neural development [7,8]. Insights into fetal metabolism of mannose and inositol have been provided by recent reports which show that, like glucose, the fetus is dependent on its mannose supply from the mother. Conversely, the fetus and placenta endogenously produce inositol from glucose [9,10]. In the term neonate, the utilization rates of mannose and inositol are much higher than the rate which could be obtained from human milk, suggesting endogenous production of both substrates after birth to meet daily requirements [11,12].

Pathological conditions in pregnancy such maternal under nutrition, placental insufficiency, and diabetes have the potential to increase or decrease the delivery of carbohydrates to the fetus and adversely affect growth, body composition, and long term outcomes in human and livestock pregnancy [13,14]. For example, diabetic pregnancies are characterized by increased glucose transfer to the fetus with subsequent elevations in fetal insulin concentrations [15]. In adults, insulin regulates mannose concentrations independent of glucose by suppression of hepatic mannose production [16-19]. Thus, the fetus is potentially at risk for hypomannosemia, both from fetal hyperinsulinemia as well as from decreased mannose delivery from the mother if insulin is used to manage maternal diabetes. Placental insufficiency in humans and sheep, on the other hand, restricts glucose delivery to the fetus leading to decreased fetal glucose and insulin concentrations [20,21]. Placental insufficiency in pregnant sheep also decreases fetal sorbitol and inositol concentrations [22]. Thus, a better understanding of the metabolism of non glucose carbohydrates during pregnancy is warranted, especially when carbohydrate supply to the fetus is compromised.

We experimentally decreased maternal glucose concentrations in sheep pregnancies using an insulin infusion both short term (2-wk) and long term (8-wk) to test the hypothesis that a maternal insulin infusion would suppress maternal and thus fetal mannose and inositol concentrations. We also used direct fetal insulin infusions in the form of a 1-wk hyperinsulinemic-isoglycemic clamp to determine the relative importance of fetal glucose and insulin concentrations for regulating fetal non glucose carbohydrates. Finally, we report for the first time the concentrations of several other carbohydrates and polyols under each of these conditions.

## Methods

### Surgical preparation

Three separate groups of Columbia-Rambouillet ewes with singleton pregnancies were used for the following experiments: 1) 2-wk maternal insulin infusion, 2) 8-wk maternal insulin infusion, and 3) 1-wk direct fetal insulin infusion. All maternal and fetal surgical preparations and post operative care have been previously described [23-25]. Maternal femoral venous and arterial catheters were placed through a left groin incision. Fetal infusion catheters were placed into fetal femoral veins via hind limb pedal veins and sampling catheters were placed into the fetal abdominal aorta via a hind limb pedal artery. Ewes were fed Premium Alfalfa Pellets (Standlee; Kimberly ID) and intake was not different between control and hypoglycemic groups (1.80 ± 0.10 kg/d control; 1.59 ± 0.10 kg/d hypoglycemic).

### Care and use of animals

All animal procedures have followed established standards for the humane care and use of animals and were in compliance with guidelines of the United States Department of Agriculture, the National Institutes of Health, and the American Association for the Accreditation of Laboratory Animal Care. The animal care and use protocols were approved by the University of Colorado Institutional Animal Care and Use Committee.

### Experimental design

#### Two-Wk maternal insulin infusion

For the 2-wk maternal insulin infusion group and their respective controls, maternal and fetal catheters were surgically placed at 122.8 ± 1.3 d of gestation (dGA; term = 148 dGA) [23,26-28]. One randomly assigned group received a continuous maternal infusion of intravenous insulin for 2-wk. Maternal arterial plasma glucose was measured at least twice daily and the insulin infusion was adjusted to achieve a 40-50% reduction in glucose concentrations (2-wk HG; n = 8). Maternal insulin concentrations are approximately doubled by this experimental design [29]. The control group received a maternal saline infusion at rates matched to the insulin infusion rates (2-wk C; n = 13). Fetal arterial plasma was sampled at the end of the 2-wk maternal infusion period for insulin and carbohydrate measurements.

#### Eight-Wk maternal insulin infusion

For the 8-wk maternal insulin group and their respective controls, an initial surgery was performed at 70.0 ± 0.8 dGA to place maternal catheters [25]. One randomly assigned group (n = 9) received a continuous maternal infusion of intravenous insulin for 8-wk. Maternal arterial plasma glucose was measured at least twice daily and the insulin infusion was adjusted to achieve a 40-50% reduction in glucose concentrations. Maternal insulin concentrations are approximately doubled by this experimental design [30,31]. The control group received a maternal saline infusion at rates matched to the insulin infusion rates (8-wk C; n = 5). At 119.4 ± 0.5 dGA, a second surgery was performed to place fetal catheters. After the second surgery, 8-wk HG ewes were further randomly divided into 2 groups. Fetuses in one of these groups received a direct fetal insulin infusion for the final week of the study (8-wk HG+I; n = 4). The insulin infusion was kept constant at 100 mU/h (using necropsy weights = 38.9 mU/kg/h ± 2.8) and ran concurrently with a direct fetal infusion of 33% dextrose (wt/vol) to prevent a further fall in fetal glucose concentrations. Fetal arterial plasma glucose concentrations were measured at least twice daily and the dextrose infusion was adjusted accordingly. The other group received a direct fetal saline infusion matched at equal infusion rates to the combined insulin and dextrose infusion (8-wk HG; n = 5). Finally, fetuses in the 8-wk C group also received a direct fetal saline infusion at equal rates. Fetal arterial plasma was sampled at the end of the infusions for insulin and carbohydrate measurements.

#### One-Wk fetal insulin infusion

Six late gestation animals were used in this experiment. Fetal catheters were placed at 118.5 ± 0.6 dGA. All fetuses received an insulin infusion with a concurrent dextrose infusion into a fetal hind limb vein. The insulin infusion rate was progressively increased such that infusion rates ranged from 36.6 ± 8.4 mU/h on the first day to 121.8 ± 1.2 mU/h on the final d. The fetal dextrose infusion was adjusted to prevent a fall in glucose concentrations based on measurement of fetal arterial plasma glucose once or twice daily. Baseline, 4d, and 7d fetal arterial plasma was sampled for insulin and carbohydrate measurements.

### Biochemical analysis

Whole blood was collected in EDTA-coated syringes and immediately centrifuged (14,000 g) for 3 min at 4°C. Plasma was removed and the glucose concentration immediately determined using the YSI model 2700 select biochemistry analyzer (Yellow Springs Instruments, Yellow Springs, OH) [23]. The remainder of the plasma was stored at −70°C for insulin and carbohydrate measurements. Insulin (Alpco; inter-assay and intra-assay coefficients of variation: 2.9 and 5.6%) was measured by enzyme-linked immunosorbent assay [23]. Plasma was analyzed for mannose, inositol, fructose, mannitol, erythritol, arabinol, sorbitol and ribitol by HPLC as we have previously described [9].

### Postmortem exam

Liver tissue from the right hepatic lobe was obtained in the 8-wk HG, 8-wk C, and 8-wk HG+I fetuses under conditions closely approximating *in vivo* study conditions as previously described for measurement of sorbitol and fructose [25].

### Statistical analysis

Statistical analysis was performed with SAS v.9.2 (SAS Institute) and GraphPad Prism 4. Results are expressed as mean ± SEM. *P*-values less than 0.05 were considered significant. The 2-wk HG and C fetuses were compared with Student’s *t* test (parametric data) or the Mann–Whitney test (non parametric data). The 8-wk HG, HG+I, and C fetuses were compared with a one-way ANOVA. For measurements taken at multiple time points within an animal a mixed models ANOVA was used with terms for experimental group, time, group by time interaction as indicated. Repeated measurements made within an animal were accounted for. Post-test comparisons were made using Fishers least squares difference if the overall ANOVA had a *P* < 0.05.

## Results

### Two-Wk maternal insulin infusion

#### Fetal insulin, carbohydrates, and polyols

As previously reported [23,26-28], gestational ages were similar, but fetal weight, fetal arterial plasma insulin, and glucose were 24%, 63%, and 49% lower, respectively in the 2 wk HG group (*P* < 0.01, Table 1). We found lower fetal arterial plasma concentrations of mannose (35%, *P* < 0.01), sorbitol (57%, *P* < 0.01), and fructose (60%, *P* < 0.01) in the 2-wk HG group (Table 1). Although the mean arterial plasma inositol concentration was nearly doubled in the 2-wk HG fetuses this did not reach statistical significance. Other fetal plasma carbohydrate concentrations were similar between 2-wk HG and C groups (Table 1).

**Table 1:** Two-Wk maternal insulin infusion

**Measurement**	**Control**	**Hypoglycemic**
Gestational age, d	138.5 ± 0.4	137.5 ± 0.8
Fetal weight, kg	4.37 ± 0.14	3.30 ± 0.18**
Fetal plasma arterial insulin, ng/mL	0.30 ± 0.03	0.11 ± 0.01**
Fetal plasma arterial carbohydrates, μmol/L		
Glucose	1,159 ± 64	594 ± 47**
Mannose	24.5 ± 1.2	15.9 ± 1.4**
Inositol	638.5 ± 138.6	1,129.0 ± 256.1
Sorbitol	123.8 ± 12.7	53.9 ± 10.5**
Fructose	4771 ± 450	1,909 ± 356**
Erythritol	399.6 ± 28.1	345.9 ± 20.8
Arabinol	245.0 ± 11.9	225.3 ± 15.6
Ribitol	128.1 ± 9.1	148.5 ± 18.8
Mannitol	72.8 ± 9.7	49.8 ± 10.1

Values are means ± SEM. ** refer to significant differences between Control (n = 13) and Hypoglycemic (n = 8); *P* < 0.01 by Students *t* test or the Mann–Whitney test.

### Eight-Wk maternal insulin infusion

#### Maternal plasma carbohydrates and polyols

Since 2-wk maternal insulin infusion experiments focused only on plasma carbohydrate changes in the fetal circulation, we included maternal carbohydrate analysis with the 8-wk insulin infusion studies. Consistent with study design, maternal arterial plasma glucose concentrations in the 8-wk HG group were approximately 40% lower compared to 8-wk C group throughout the insulin infusion period as previously reported (Table 2, *P* < 0.01) [25]. Maternal arterial plasma mannose concentrations also were 48% lower in the 8 wk HG group (Table 2, *P* < 0.01), but maternal arterial plasma inositol, sorbitol, erythritol, arabinol, and ribitol did not change (Table 2).

**Table 2:** Eight-Wk maternal insulin infusion

**Measurement**	**Control**	**Hypoglycemic**	**HG+I**
Gestational age, d	133.2 ± 1.1	133.0 ± 1.3	134.5 ± 0.9
Maternal plasma arterial carbohydrates, μmol/L			
Glucose	3,605 ± 203	1,715 ± 145**	1,817 ± 95**
Mannose	67.0 ± 4.3	34.7 ± 3.5**	31.4 ± 5.0**
Inositol	27.7 ± 4.4	34.9 ± 3.9	34.6 ± 3.1
Sorbitol	13.4 ± 3.4	18.8 ± 4.2	11.8 ± 1.3
Erythritol	52.8 ± 5.5	44.5 ± 3.1	39.6 ± 8.5
Arabinol	96.2 ± 13.3	125.6 ± 6.5	118.0 ± 23.3
Ribitol	25.6 ± 3.0	25.9 ± 3.2	16.1 ± 4.3
Fetal weight, kg	3.66 ± 0.12	2.20 ± 0.14**	2.61 ± 0.21**
Fetal plasma arterial insulin, ng/mL	0.46 ± 0.09	0.16 ± 0.03*	0.66 ± 0.17
Fetal plasma arterial carbohydrates, μmol/L			
Glucose	1,553 ± 282	587 ± 56**	511 ± 37**
Mannose	30.4 ± 3.8	17.0 ± 1.1**	7.9 ± 0.9**
Inositol	741.4 ± 240.8	910.1 ± 157.5	792.2 ± 199.5
Sorbitol	188.5 ± 31.9	55.2 ± 11.9**	41.3 ± 8.3**
Fructose	4,508 ± 981	1,631 ± 301**	800 ± 154**
Erythritol	394.6 ± 30.7	372.2 ± 9.3	311.6 ± 24.6
Arabinol	235.3 ± 17.1	237.5 ± 15.1	214.9 ± 54.3
Ribitol	124.3 ± 15.9	139.6 ± 21.7	156.8 ± 57.6
Mannitol	47.4 ± 8.5	31.4 ± 6.1	33.8 ± 8.7
Fetal hepatic sorbitol and fructose, nmol/g			
Sorbitol	589.0 ± 90.1	205.8 ± 42.1**	161.3 ± 56.0**
Fructose	1,679 ± 219	398 ± 119**	215 ± 95**

Values are means ± SEM. *, ** refer to significant differences from Control (n = 5) animals; *P* < 0.05, 0.01, respectively, by ANOVA.

#### Fetal insulin, carbohydrates, and polyols

**Figure 1:**
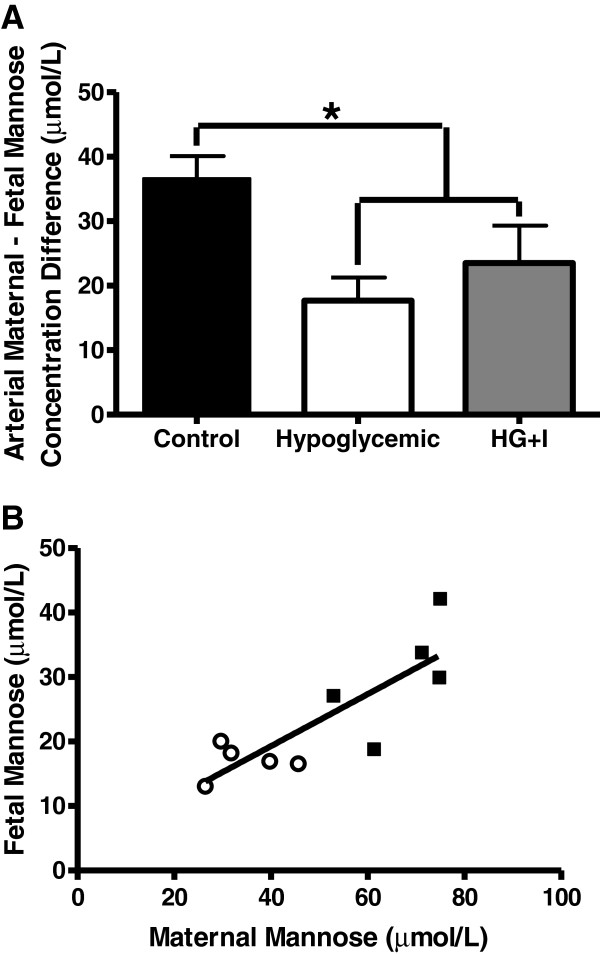
**Fetal and maternal mannose concentrations following an eight wk maternal insulin infusion. A)** The maternal-fetal arterial plasma mannose difference measured in control (n = 5), hypoglycemic (n = 5), and hypoglycemic + insulin (HG + I, n = 4).* indicates *P* < 0.05. Values are mean ± SEM. **B)** In both control (black squares, n = 5) and hypoglycemic fetal sheep (open circles, n = 5) fetal and maternal arterial plasma mannose concentrations are highly correlated in control (black squares) hypoglycemic animals (r^2^ = 0.69, *P* < 0.01).

As previously reported, gestational age at the time of study was not different between the groups. Fetal weights were 40% lower in the 8-wk HG group compared to 8-wk C (Table 2, *P* < 0.01) [25]. Fetal arterial plasma insulin and glucose were lower in the 8-wk HG group (*P* < 0.05, Table 2) [25]. Fetal arterial plasma mannose was 44% lower (*P* < 0.01), as were sorbitol (71%, *P* < 0.01) and fructose (64%, *P* < 0.01, Table 2). Furthermore, the arterial plasma maternal - fetal mannose difference also was lower (*P* < 0.01, Figure 1A), and fetal and maternal arterial plasma mannose concentrations were highly correlated (Figure 1B). Fetal arterial plasma inositol, erythritol, arabinol, ribitol, and mannitol were similar between 8-wk HG and C groups (Table 2).

A direct fetal insulin infusion for the final wk of the 8-wk maternal insulin infusion with a concurrent direct fetal dextrose infusion to prevent a further fall in fetal arterial plasma glucose was used to determine the effect of fetal glucose and insulin concentrations on regulating fetal non glucose carbohydrates. The direct fetal insulin infusion with a concurrent dextrose infusion (HG+I) resulted in fetal arterial plasma insulin concentrations 3-fold higher than HG fetuses (*P* < 0.05) without a change in fetal arterial plasma glucose concentrations (Table 2), as previously reported [25]. HG+I fetuses demonstrated even lower arterial plasma mannose concentrations when compared to the HG group (53%, *P* < 0.05), and had mean fructose concentrations which were over 50% lower, though this failed to reach statistical significance (*P* = 0.058 by post hoc Student’s *t* test). There was no effect on fetal arterial plasma inositol, sorbitol, erythritol, arabinol, ribitol, and mannitol (Table 2). Fetal hepatic sorbitol and fructose concentrations were lower in the 8-wk HG group compared to C fetal livers (*P* < 0.01, Table 2). HG+I fetuses did not have changes in hepatic sorbitol or fructose compared to the 8-wk HG fetuses (Table 2).

### One-Wk fetal insulin infusion

In order to determine the effect of fetal glucose and insulin concentrations for regulating non glucose carbohydrates in fetuses whose mothers did not receive a chronic insulin infusion, we infused 6 fetuses directly with insulin with a concurrent dextrose infusion to maintain fetal glucose concentrations for 1 wk beginning on 125.2 ± 0.7 dGA. Fetal plasma arterial glucose concentrations were stable throughout the infusion period, however the dextrose infusion was increased progressively during the insulin infusion period to maintain euglycemia, starting at 3.6 ± 1.1 mg/min and ending at 12.1 ± 2.5 mg/min. Fetal arterial plasma insulin, carbohydrate, and polyol concentrations were measured at baseline and on d 4 and d 7 of the insulin infusion (Table 3). Fetal arterial plasma insulin concentrations were increased (*P* < 0.01) and fetal arterial plasma mannose, fructose, and erythritol concentrations were decreased (*P* < 0.05).

**Table 3:** One-Wk fetal insulin infusion

**Measurement**	**d0**	**d4**	**d7**
Fetal plasma arterial insulin, ng/mL	0.26 ± 0.03	0.92 ± 0.20**	0.92 ± 0.10**
Fetal plasma arterial carbohydrates, μmol/L			
Glucose	1,338 ± 96	998 ± 76	1,201 ± 79
Mannose	33.9 ± 2.1	18.9 ± 2.4**	16.8 ± 2.1**
Inositol	838.2 ± 165.2	703.1 ± 144.3	673.3 ± 85.0
Sorbitol	163.4 ± 62.7	134.2 ± 76.1	82.5 ± 27.1
Fructose	5,751 ± 884	3,214 ± 673**	2,658 ± 538**
Erythritol	402.1 ± 38.2	356.5 ± 32.2*	330.3 ± 32.0**
Arabinol	193.9 ± 24.1	176.5 ± 28.4	178.3 ± 23.8
Ribitol	153.1 ± 21.4	148.9 ± 22.0	133.9 ± 17.1
Mannitol	69.4 ± 5.8	64.5 ± 10.0	60.1 ± 8.8

Values are means ± SEM. *, ** refer to significant differences from d0; *P* < 0.05, 0.01, respectively, by ANOVA. n = 6.

## Discussion

Non glucose carbohydrates are important for normal fetal development [1-4]. Pathological conditions in pregnancy that adversely affect glucose delivery to the fetus could affect the delivery of non glucose carbohydrates. Therefore, the primary goal of the current study was to determine how a maternal insulin infusion with an associated reduction in glucose supply to the fetus affected the plasma concentrations of these metabolites in both the maternal and fetal circulations. We found that a maternal insulin infusion which reduced glucose supply to the fetus of both short (2-wk) and long (8-wk) durations resulted in decreased maternal and fetal mannose concentrations along with decreased fetal concentrations of sorbitol and fructose. As in human pregnancies, fetal and maternal arterial plasma mannose concentrations were highly correlated, suggesting that maternal mannose concentration determines fetal mannose concentrations. However, a physiological increase in fetal insulin after prolonged fetal hypoglycemia further reduced circulating fetal mannose concentrations, indicating that insulin also plays a key role in regulating mannose concentrations. Finally, we found that neither maternal nor fetal arterial plasma inositol concentrations changed with exogenous insulin infusions.

The most striking findings in this study relate to the regulation of fetal mannose concentrations. There has been emerging evidence in both normal human and sheep pregnancies that the fetus is dependent on placental delivery of mannose [9,10,32]. In fact, in human pregnancies, it appears that over 95% of fetal circulating mannose is derived from transplacental transfer from the mother [10]. Our findings also support transplacental transfer of mannose from the mother as the dominant source of circulating fetal mannose, as we found that maternal and fetal plasma arterial mannose concentrations were highly correlated when an experimental maternal insulin infusion decreased maternal mannose concentrations. Furthermore, we found a significant decrease in the maternal to fetal mannose concentration difference in 8-wk HG sheep. This finding suggests that decreased fetal mannose concentrations were due to decreased maternal mannose concentrations rather than a decrease in placental transport capacity. In fact, when fetal and maternal mannose concentrations were measured in a sheep model of chronic placental insufficiency there was a significant increase in the maternal to fetal mannose concentration gradient, indicating that placental insufficiency has the potential to disrupt maternal to fetal mannose delivery [22].

Our results also demonstrate a key role for insulin in the regulation of both maternal and fetal plasma mannose concentrations. We report for the first time that long term (8-wk) maternal insulin infusion during pregnancy results in a 50% reduction in maternal mannose concentrations. As these maternal ewes were also chronically hypoglycemic, we cannot determine whether chronic hypoglycemia or chronic hyperinsulinemia was directly responsible for reductions in mannose concentrations. However, previous work in rodents and adults has shown that insulin is independently involved in lowering plasma mannose by suppressing hepatic glycogen breakdown and mannose efflux from the liver [16,18]. In healthy adults, oral glucose administration increased both glucose and insulin concentrations yet mannose concentrations were still decreased, arguing for insulin stimulated mannose disposal independent of glucose [17,19].

From the present study, however, we were able to gain some insight into the independent effects of glucose and insulin on circulating mannose in the fetus. When a direct fetal insulin infusion restored physiological insulin concentrations in 8-wk HG+I fetuses and fetal glucose concentrations were maintained, fetal mannose concentrations were further reduced by 50%. This argues for insulin mediated reductions in fetal plasma mannose to extremely low concentrations (~8 μmol/L) independent of concurrent hypoglycemia. To further determine the independent effects of glucose and insulin on fetal mannose, we infused insulin with a concurrent dextrose infusion to maintain glucose concentrations in a separate group of normal late gestation fetuses. This fetal hyperinsulinemic-euglycemic clamp also resulted in decreased mannose concentrations, confirming a role for insulin in the regulation of fetal mannose independent of glucose concentrations.

We also showed decreased fetal arterial plasma sorbitol and fructose concentrations in 8-wk HG fetuses. Contrary to our hypothesis, fetal plasma inositol concentrations were maintained during restricted fetal glucose supply. Maternal glucose has several potential fates once it enters the placenta. It can be directly transferred to the fetus or oxidized for fuel production [10,33]. Additionally, the placenta can convert glucose to inositol or sorbitol [9,22,32]. Glucose is converted to inositol by glucose-6-phosphate:1-phosphate cyclase and to sorbitol by aldose reductase [34-36]. The balance between these two pathways is regulated by NADPH derived from the placental uptake of glutamate from the fetus [37-40]. Although we did not directly measure placental glutamate uptake in this study, fetal arterial plasma glutamate concentrations are reduced by 50% in HG fetuses, which might lead to a reduction in placental NADPH availability [41]. This would, in turn, limit placental production of sorbitol and preserve or increase the production of inositol. This also is consistent with our findings of decreased fetal plasma fructose and decreased hepatic sorbitol and fructose, as the major fate for fetal sorbitol in the sheep is conversion to fructose in the liver by sorbitol dehydrogenase.

Interestingly, fetal fructose concentrations also are decreased by fetal insulin infusion independent of glucose. Relationships between fructose and insulin have been previously reported, such that pancreatectomized fetal sheep have increased fructose concentrations [42], and fructose infusion into the sheep fetus can stimulate insulin secretion [43]. The role of fructose in human fetal and neonatal development remains to be determined, though postulated roles include alternative pathways in glucose metabolism, redox balance, and lipid synthesis [6,44].

The results of our study are limited only to changes in maternal and fetal arterial plasma concentrations of carbohydrates and polyols, thus conclusions cannot be made regarding their uptake and utilization rates by the fetus. However, the results are consistent with previous studies in human fetuses showing placental transport of maternal mannose, fetal production of inositol, and placental export to the fetus of sorbitol [9,10]. Future studies are warranted to determine the effects of fetal glucose and insulin on uptake, production, and utilization rates of these carbohydrates, thus providing a more in depth understanding of their metabolism in the pregnant mother and fetus.

## Conclusions

In summary, the results of this study show that a chronic and constant maternal insulin infusion suppresses both maternal and fetal mannose concentrations by approximately 50%. Additionally, insulin can suppress fetal mannose and fructose concentrations independent of glucose availability. The functional implications of this degree of fetal hypomannosemia are unclear, but a recent report demonstrating embryonic lethality in mice with a hypomorphic phosphomannomutase 2 gene defect shows the critical role of mannose in normal fetal development [4]. Our results also show a significant reduction in fetal sorbitol concentrations, likely due to decreased placental sorbitol production and transfer to the fetus following increased shuttling of placental glucose into inositol production. Taken together, our findings demonstrate the potential for other carbohydrates, in addition to glucose, to be adversely affected by alterations in maternal and/or fetal insulin concentrations and glucose supply. Given the important role that many of these non glucose carbohydrates have in fetal development, future research on pathological conditions in pregnancy should include further investigation into carbohydrate metabolism beyond glucose, especially when conditions of fetal hyperinsulinemia are considered.

## Abbreviations

dGA: Days gestational age; HG: Hypoglycemic; C: Control; HG+I: Iypoglycemic plus insulin.

## Competing interests

The authors declare that they have no competing interests.

## Authors’ contributions

LDB and PJR conceived of the study, designed the study, acquired and interpreted data, and wrote the first draft of the manuscript. ST conceived of the study, acquired and interpreted data, and reviewed the manuscript. JL acquired and interpreted data and reviewed the manuscript. AC designed the study, acquired and interpreted data, and reviewed the manuscript. FB conceived of the study, designed the study, interpreted data, and reviewed the manuscript. All authors read and approved the final manuscript.

## References

[B1] Groenen PM Peer PG Wevers RA Swinkels DW Franke B Mariman EC Steegers-Theunissen RP Maternal myo-inositol, glucose, and zinc status is associated with the risk of offspring with spina bifida Am J Obstet Gynecol 2003 189 1713 1719 10.1016/S0002-9378(03)00807-X 14710103

[B2] Hallman M Saugstad OD Porreco RP Epstein BL Gluck L Role of myoinositol in regulation of surfactant phospholipids in the newborn Early Hum Dev 1985 10 245 254 10.1016/0378-3782(85)90055-6 3838720

[B3] Reece EA Khandelwal M Wu YK Borenstein M Dietary intake of myo-inositol and neural tube defects in offspring of diabetic rats Am J Obstet Gynecol 1997 176 536 539 10.1016/S0002-9378(97)70543-X 9077602

[B4] Schneider A Thiel C Rindermann J DeRossi C Popovici D Hoffmann GF Grone HJ Korner C Successful prenatal mannose treatment for congenital disorder of glycosylation-Ia in mice Nat Med 2012 18 71 73 22157680 10.1038/nm.2548

[B5] Davis JA Freeze HH Studies of mannose metabolism and effects of long-term mannose ingestion in the mouse Biochim Biophys Acta 2001 1528 116 126 10.1016/S0304-4165(01)00183-0 11687298

[B6] Trindade CE Barreiros RC Kurokawa C Bossolan G Fructose in fetal cord blood and its relationship with maternal and 48-hour-newborn blood concentrations Early Hum Dev 2011 87 193 197 10.1016/j.earlhumdev.2010.12.005 21276669

[B7] Greene ND Copp AJ Mouse models of neural tube defects: investigating preventive mechanisms Am J Med Genet C: Semin Med Genet 2005 135C 31 41 10.1002/ajmg.c.30051 15800852

[B8] Jaeken J Matthijs G Congenital disorders of glycosylation: a rapidly expanding disease family Annu Rev Genomics Hum Genet 2007 8 261 278 10.1146/annurev.genom.8.080706.092327 17506657

[B9] Brusati V Jozwik M Jozwik M Teng C Paolini C Marconi AM Battaglia FC Fetal and maternal non-glucose carbohydrates and polyols concentrations in normal human pregnancies at term Pediatr Res 2005 58 700 704 10.1203/01.PDR.0000180549.86614.73 16189196

[B10] Staat BC Galan HL Harwood JE Lee G Marconi AM Paolini CL Cheung A Battaglia FC Transplacental supply of mannose and inositol in uncomplicated pregnancies using stable isotopes J Clin Endocrinol Metab 2012 97 2497 2502 10.1210/jc.2011-1800 22544916 PMC3387389

[B11] Cavalli C Teng C Battaglia FC Bevilacqua G Free sugar and sugar alcohol concentrations in human breast milk J Pediatr Gastroenterol Nutr 2006 42 215 221 10.1097/01.mpg.0000189341.38634.77 16456418

[B12] Brown LD Cheung A Harwood JE Battaglia FC Inositol and mannose utilization rates in term and late-preterm infants exceed nutritional intakes J Nutr 2009 139 1648 1652 10.3945/jn.109.109108 19494026 PMC2728690

[B13] De Blasio MJ Gatford KL McMillen IC Robinson JS Owens JA Placental restriction of fetal growth increases insulin action, growth, and adiposity in the young lamb Endocrinology 2007 148 1350 1358 10.1210/en.2006-0653 17110432

[B14] Long NM Tousley CB Underwood KR Paisley SI Means WJ Hess BW Du M Ford SP Effects of early- to mid-gestational undernutrition with or without protein supplementation on offspring growth, carcass characteristics, and adipocyte size in beef cattle J Anim Sci 2012 90 197 206 21908644 10.2527/jas.2011-4237

[B15] Schwartz R Gruppuso PA Petzold K Brambilla D Hiilesmaa V Teramo KA Hyperinsulinemia and macrosomia in the fetus of the diabetic mother Diabetes Care 1994 17 640 648 10.2337/diacare.17.7.640 7924772

[B16] Sharma V Freeze HH Mannose efflux from the cells: a potential source of mannose in blood J Biol Chem 2011 286 10193 10200 10.1074/jbc.M110.194241 21273394 PMC3060472

[B17] Sone H Shimano H Ebinuma H Takahashi A Yano Y Iida KT Suzuki H Toyoshima H Kawakami Y Okuda Y Noguchi Y Ushizawa K Saito K Yamada N Physiological changes in circulating mannose levels in normal, glucose-intolerant, and diabetic subjects Metabolism 2003 52 1019 1027 10.1016/S0026-0495(03)00153-7 12898467

[B18] Taguchi T Yamashita E Mizutani T Nakajima H Yabuuchi M Asano N Miwa I Hepatic glycogen breakdown is implicated in the maintenance of plasma mannose concentration Am J Physiol Endocrinol Metab 2005 288 E534 E540 15536204 10.1152/ajpendo.00451.2004

[B19] Wood FC Jr Cahill GF Jr Mannose utilization in man J Clin Invest 1963 42 1300 1312 10.1172/JCI104814 14057858 PMC289400

[B20] Nicolini U Hubinont C Santolaya J Fisk NM Rodeck CH Effects of fetal intravenous glucose challenge in normal and growth retarded fetuses Horm Metab Res 1990 22 426 430 10.1055/s-2007-1004939 2227801

[B21] Limesand SW Rozance PJ Smith D Hay WW Jr Increased insulin sensitivity and maintenance of glucose utilization rates in fetal sheep with placental insufficiency and intrauterine growth restriction Am J Physiol Endocrinol Metab 2007 293 E1716 E1725 10.1152/ajpendo.00459.2007 17895285

[B22] Regnault TR Teng C de Vrijer B Galan HL Wilkening RB Battaglia FC The tissue and plasma concentration of polyols and sugars in sheep intrauterine growth retardation Exp Biol Med (Maywood) 2010 235 999 1006 10.1258/ebm.2010.009360 20576742

[B23] Rozance PJ Limesand SW Hay WW Jr Decreased nutrient-stimulated insulin secretion in chronically hypoglycemic late-gestation fetal sheep is due to an intrinsic islet defect Am J Physiol Endocrinol Metab 2006 291 E404 E411 10.1152/ajpendo.00643.2005 16569758

[B24] Maliszewski AM Gadhia MM O’Meara MC Thorn SR Rozance PJ Brown LD Prolonged infusion of amino acids increases leucine oxidation in fetal sheep Am J Physiol Endocrinol Metab 2012 302 E1483 E1492 10.1152/ajpendo.00026.2012 22454287 PMC3378157

[B25] Thorn SR Sekar SM Lavezzi JR O’Meara MC Brown LD Hay WW Jr Rozance PJ A physiological increase in insulin suppresses gluconeogenic gene activation in fetal sheep with sustained hypoglycemia Am J Physiol Regul Integr Comp Physiol 2012 303 R861 R869 10.1152/ajpregu.00331.2012 22933022 PMC3469666

[B26] Limesand SW Rozance PJ Brown LD Hay WW Jr Effects of chronic hypoglycemia and euglycemic correction on lysine metabolism in fetal sheep Am J Physiol Endocrinol Metab 2009 296 E879 E887 10.1152/ajpendo.90832.2008 19190258 PMC2670627

[B27] Rozance PJ Limesand SW Zerbe GO Hay WW Jr Chronic fetal hypoglycemia inhibits the later steps of stimulus-secretion coupling in pancreatic beta-cells Am J Physiol Endocrinol Metab 2007 292 E1256 E1264 10.1152/ajpendo.00265.2006 17213478

[B28] Thorn SR Regnault TRH Brown LD Rozance PJ Keng J Roper M Wilkening RB Hay WW Jr Friedman JE Intrauterine growth restriction increases fetal hepatic gluconeogenic capacity and reduces messenger ribonucleic acid translation initiation and nutrient sensing in fetal liver and skeletal muscle Endocrinology 2009 150 3021 3030 10.1210/en.2008-1789 19342452 PMC2703533

[B29] DiGiacomo JE Hay WW Jr Fetal glucose metabolism and oxygen consumption during sustained hypoglycemia Metabolism 1990 39 193 202 10.1016/0026-0495(90)90075-N 2405236

[B30] Carver TD Hay WW Jr Uteroplacental carbon substrate metabolism and O2 consumption after long-term hypoglycemia in pregnant sheep Am J Physiol 1995 269 E299 E308 7653547 10.1152/ajpendo.1995.269.2.E299

[B31] Carver TD Quick AA Teng CC Pike AW Fennessey PV Hay WW Jr Leucine metabolism in chronically hypoglycemic hypoinsulinemic growth-restricted fetal sheep Am J Physiol Endocrinol Metab 1997 272 E107 E117 10.1152/ajpendo.1997.272.1.E107 9038859

[B32] Teng CC Tjoa S Fennessey PV Wilkening RB Battaglia FC Transplacental carbohydrate and sugar alcohol concentrations and their uptakes in ovine pregnancy Exp Biol Med (Maywood) 2002 227 189 195 11856817 10.1177/153537020222700306

[B33] Aldoretta PW Hay WW Jr Effect of glucose supply on ovine uteroplacental glucose metabolism Am J Physiol Regul Integr Comp Physiol 1999 277 R947 R958 10.1152/ajpregu.1999.277.4.R947 10516231

[B34] Brachet EA Presence of the complete sorbitol pathway in the human normal umbilical cord tissue Biol Neonate 1973 23 314 323 10.1159/000240609 4149330

[B35] Mango D Scirpa P Menini E Effects of dehydroepiandrosterone and 16 alpha-hydroxydehydroepiandrosterone on the reduction of glucose to glucitol by the human placenta Horm Metab Res 1976 8 302 307 10.1055/s-0028-1093641 133979

[B36] Quirk JG Jr Bleasdale JE Myo-inositol homeostasis in the human fetus Obstet Gynecol 1983 62 41 44 6687929

[B37] Ginsburg J Jeacock MK Pathways of glucose metabolism in human placental tissue Biochim Biophys Acta 1964 90 166 168 10.1016/0304-4165(64)90130-8 14201151

[B38] Makarewicz W Swierczynski J Phosphate-dependent glutaminase in the human term placental mitochondria Biochem Med Metab Biol 1988 39 273 278 10.1016/0885-4505(88)90085-0 3395507

[B39] Moores RR Jr Vaughn PR Battaglia FC Fennessey PV Wilkening RB Meschia G Glutamate metabolism in fetus and placenta of late-gestation sheep Am J Physiol 1994 267 R89 R96 7914073 10.1152/ajpregu.1994.267.1.R89

[B40] Sakurai T Takagi H Hosoya N Metabolic pathways of glucose in human placenta. Changes with gestation and with added 17-beta-estradiol Am J Obstet Gynecol 1969 105 1044 1054 5352584 10.1016/0002-9378(69)90125-2

[B41] Limesand SW Hay WW Jr Adaptation of ovine fetal pancreatic insulin secretion to chronic hypoglycaemia and euglycaemic correction J Physiol Lond 2003 547 95 105 10.1113/jphysiol.2002.026831 12562941 PMC2342612

[B42] Fowden AL Comline RS The effects of pancreatectomy on the sheep fetus in utero Q J Exp Physiol 1984 69 319 330 6374729 10.1113/expphysiol.1984.sp002808

[B43] Philipps AF Carson BS Meschia G Battaglia FC Insulin secretion in fetal and newborn sheep Am J Physiol Endocrinol Metab 1978 235 E467 E474 10.1152/ajpendo.1978.235.5.E467 364995

[B44] Meznarich HK Hay WW Jr Sparks JW Meschia G Battaglia FC Fructose disposal and oxidation rates in the ovine fetus Q J Exp Physiol 1987 72 617 625 3122263 10.1113/expphysiol.1987.sp003102

